# Causal Relationship Between Gut Microbiota, Metabolites, and Sarcopenia: A Mendelian Randomization Study

**DOI:** 10.1093/gerona/glae173

**Published:** 2024-07-12

**Authors:** Xiangyu Zhang, Guang Yang, Shide Jiang, Bingzhou Ji, Wenqing Xie, Hengzhen Li, Jianfeng Sun, Yusheng Li

**Affiliations:** Department of Orthopedics, Xiangya Hospital, Central South University, Changsha, China; Department of Orthopedics, Xiangya Hospital, Central South University, Changsha, China; National Clinical Research Center for Geriatric Disorders, Xiangya Hospital, Central South University, Changsha, China; Department of Orthopedics, The Central Hospital of Yongzhou, Yongzhou, China; Department of Orthopedics, Xiangya Hospital, Central South University, Changsha, China; National Clinical Research Center for Geriatric Disorders, Xiangya Hospital, Central South University, Changsha, China; Department of Orthopedics, Xiangya Hospital, Central South University, Changsha, China; National Clinical Research Center for Geriatric Disorders, Xiangya Hospital, Central South University, Changsha, China; Department of Orthopedics, Xiangya Hospital, Central South University, Changsha, China; National Clinical Research Center for Geriatric Disorders, Xiangya Hospital, Central South University, Changsha, China; Department of Orthopedics, Xiangya Hospital, Central South University, Changsha, China; National Clinical Research Center for Geriatric Disorders, Xiangya Hospital, Central South University, Changsha, China; Department of Orthopedics, Xiangya Hospital, Central South University, Changsha, China; National Clinical Research Center for Geriatric Disorders, Xiangya Hospital, Central South University, Changsha, China

**Keywords:** Gastrointestinal microbiome, Mendelian randomization, Sarcopenia

## Abstract

**Background:**

Gut microbiota imbalance and sarcopenia are frequently observed in older adults. Gut microbiota and their metabolites are considered risk factors contributing to the heightened risk of sarcopenia, but whether these associations are causal remains unclear.

**Methods:**

We conducted linkage disequilibrium score regression and 2-sample Mendelian randomization (MR) methods with single-nucleotide polymorphisms sourced from large-scale genome-wide association studies as instrumental variables to examine the causal associations linking gut microbiota with their metabolites to the sarcopenia. Following the MR analysis, subsequent sensitivity analyses were conducted to reinforce the robustness and credibility of the obtained results.

**Results:**

MR analysis yielded compelling evidence demonstrating the correlation between genetically predicted gut microbiota and metabolites and the risk of sarcopenia. The abundance of *Porphyromonadaceae*, *Rikenellaceae*, *Terrisporobacter*, and *Victivallis* was found to be associated with walking pace. Our study also found suggestive associations of 12 intestinal bacteria with appendicular lean mass, and of *Streptococcaceae*, *Intestinibacter*, *Paraprevotella*, *Ruminococcaceae UCG009*, and *Sutterella* with grip strength. Specifically, we identified 21 gut microbiota-derived metabolites that may be associated with the risk of sarcopenia.

**Conclusions:**

Utilizing a 2-sample MR approach, our study elucidates the causal interplay among gut microbiota, gut microbiota-derived metabolites, and the occurrence of sarcopenia. These findings suggest that gut microbiota and metabolites may represent a potential underlying risk factor for sarcopenia, and offer the promise of novel therapeutic focal points.

## Graphical Abstract

**Graphical Abstract:**
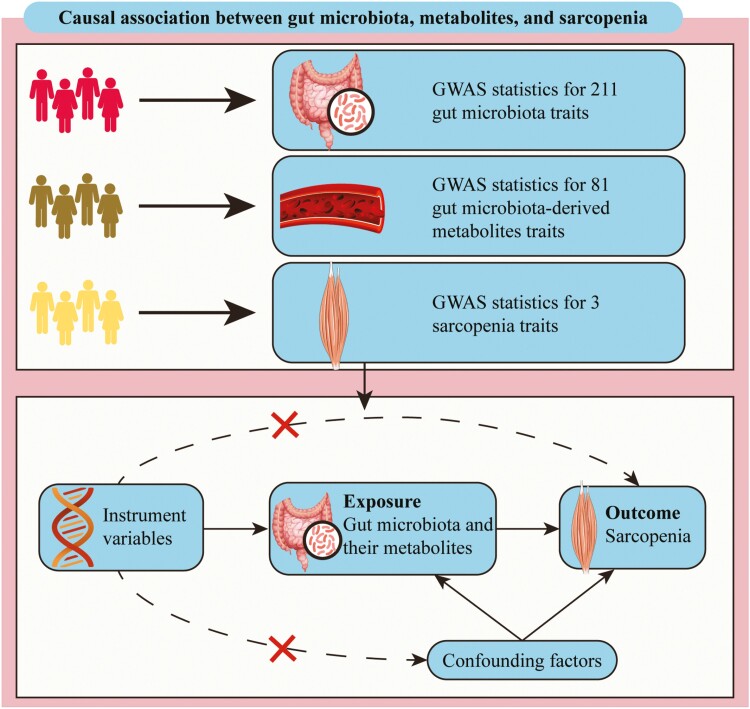

Sarcopenia is a degenerative and systemic condition that results in the loss of skeletal muscle mass and function, especially in the elderly population (1). Based on a comprehensive review and estimation of multiple research studies, it is evident that sarcopenia affects approximately 10%–16% of the global elderly population (2). Sarcopenia is intricately linked to an increased susceptibility to adverse events, including impaired functional capacity, falls, fractures, osteoporosis, metabolic syndrome, and diabetes (1,3,4). With the rapid progression of population aging, it is imperative to remain cautious of the escalating burden that sarcopenia imposes on healthcare and public health systems. About the cause of sarcopenia, there is a paucity of research elucidating its risk factors, underscoring the imperative for further exploration and uncovering (5). Evidence suggests that the composition and diversity of the gut microbiota may be determinants of skeletal muscle mass and function (6). Extensive research has already discovered the dysbiosis of the gut microbiota in populations with sarcopenia. And supplemental probiotics and fecal microbial transplantation have been shown to have therapeutic potential for sarcopenia in animal models (7,8). The gut–muscle axis suggests a complex interaction between gut microbiota and sarcopenia. However, there is still ample room for further exploration of its mechanisms and applications.

Compared to healthy individuals, patients with sarcopenia exhibit significant alterations in the composition and functionality of their gut microbiota (9). Gut dysbiosis primarily disrupts metabolic pathways, including resistance to protein synthesis and downregulation of mitochondrial energy metabolism genes. Simultaneously, gut microbiota imbalance compromises the intestinal barrier, leading to the production of byproducts such as lipopolysaccharides and pro-inflammatory cytokines, resulting in chronic inflammation and immune dysfunction (10). Furthermore, the metabolites resulting from the interaction between gut microbiota and the host affect muscle synthesis, with the absence of beneficial metabolites and an increase in harmful byproducts observed following dysbiosis (11). These mechanisms may underlie the connection between the gut–muscle axis. However, the evidence establishing a causal relationship between gut microbiota and sarcopenia is limited. And the definition of sarcopenia has undergone evolution, resulting in varying cutoff criteria across different regions over the past decade (1,12). Results from observational studies and meta-analyses should be interpreted with caution due to the presence of residual confounding and measurement errors, which may introduce bias and contribute to the uncertainty surrounding causal relationships.

Mendelian randomization (MR) is an advanced epidemiological tool that utilizes genome-wide sequencing data to investigate causal relationships. By using genetic variants as instrumental variables, MR replaces traditional exposures and evaluates their causal associations with outcomes (13). The MR design offers the advantage of providing scientific evidence comparable to that of randomized controlled trials. Significantly, the implementation of MR design circumvents the issue of reverse causation bias, as sarcopenia lacks the capability to exert an influence on an individual’s genotype. Utilizing a 2-sample MR methodology, our research capitalizes on the largest genome-wide association study (GWAS) summary statistics to date. This robust approach facilitates the exploration of causal linkages among gut microbiota, metabolites, and phenotypes associated with sarcopenia.

## Method

### Data Sources and Study Participants

We selected the most comprehensive genetic data on gut microbiota available to date, which were obtained from the MiBioGen consortium. The data set of gut microbiota was obtained from a total of 18 340 participants, encompassing individuals of European, Hispanic, and Asian descent, among others. The study further categorized bacteria at the genus level and estimated the influence of human genetics on the presence and abundance of individual microbial taxa (14). Considering that gut microbiota-derived metabolites may play a more direct role in the interaction between bacteria and the host, we have incorporated gut microbiota-derived metabolites into our research. We acquired genetic data for 81 gut microbiota-dependent metabolite features from the Human Metabolome Database (HMDB) by utilizing a comprehensive GWAS summary data set that included data from TwinsUK and KORA, with a total of 7 824 participants (15,16).

Due to the lack of unified diagnostic criteria for sarcopenia, we have selected prevalent characteristics that can serve as representative indicators, encompassing walking pace (WP), appendicular lean mass (ALM), and low grip strength (GS). The GWAS on WP was derived from data on self-reported WP from 450 967 European ancestry participants in the UK Biobank (17). The UK Biobank database has amassed GWAS data from 450 243 individuals pertaining to ALM (18). The GWAS summary data for GS were derived from a meta-analysis of 256 523 individuals aged 60 years or older across 22 cohorts comprising individuals of European descent (19). Leveraging these population-based data sets, we conducted a genetic association study on variables associated with sarcopenia within the European population.

**Figure 1.**
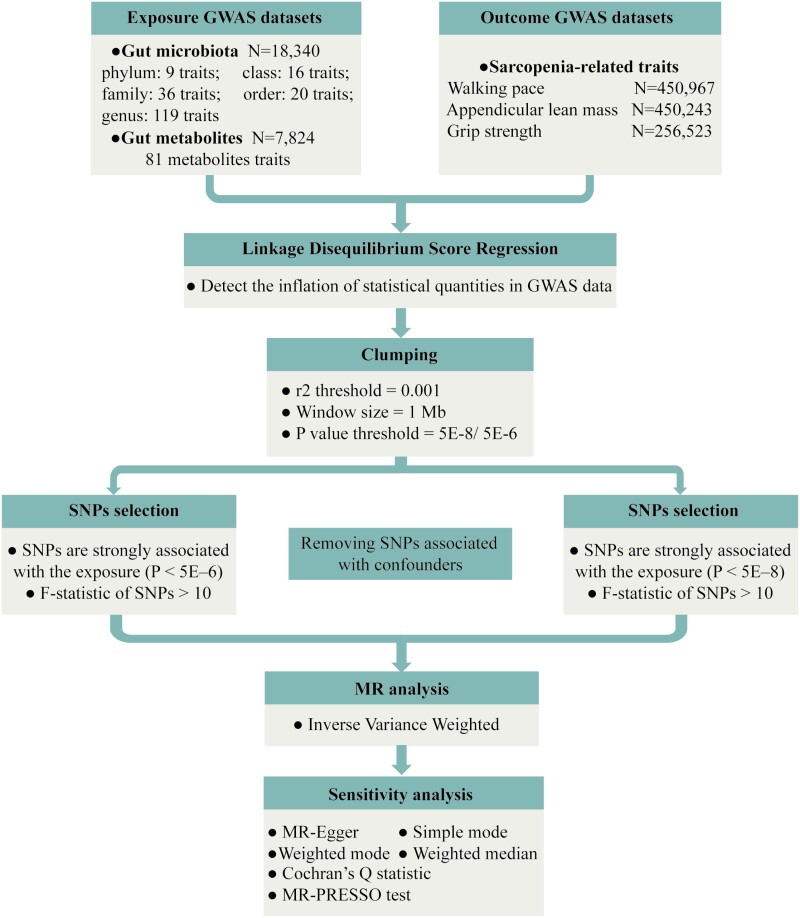
Flowchart for the study. GWAS = genome-wide association study; MR = Mendelian randomization; SNP = single-nucleotide polymorphism.

With the provided data, we utilized linkage disequilibrium score regression (LDSC) for quality control of GWAS summary data to detect the inflation of statistical quantities in GWAS data and ensure the reliability of our results (20) (Supplementary Tables 6 and 7). We conducted a 2-sample MR study to examine the causal connections between gut microbiota, metabolites, and 3 sarcopenia characteristics. The genetic variations under analysis must satisfy 3 crucial assumptions: (i) they exhibit an association with the exposure; (ii) they remain independent of confounding factors, unaffected by them; (iii) they exclusively relate to the outcome through the exposure and do not involve any other causal pathways (21). The flowchart of the study plan is shown in Figure 1.

### Genetic Instrument Selection

According to the 3 requirements of MR analysis, independent single-nucleotide polymorphisms (SNPs) associated with the exposure at a genome-wide significance level (*p* < 5 × 10^−8^) were chosen as instrumental SNPs (in LD *r*^2^ = 0.001 and kb = 10 000) (13). The corresponding association values for these SNPs in the outcome data were obtained from their respective GWAS summary statistics. However, we observed that under this restriction, the majority of exposures had only 1 SNP available for study analysis, which could introduce errors. Therefore, we relaxed the criteria for SNP selection to 5 × 10^−6^ and conducted the analysis again (Supplementary Tables 1–3). The results obtained under these 2 different criteria were subsequently harmonized to generate the final results. Subsequently, we computed the *F*-statistic to assess the strength of the selected SNPs. A genetic SNP with an *F*-statistic >10 indicates good instrument strength, which can mitigate potential bias in MR analysis. For the second assumption of MR, we searched the Phenoscanner database (http://www.phenoscanner.medschl.cam.ac.uk/) to identify whether there were SNPs associated with confounding factors (body mass index, diabetes).

### MR Analyses

The inverse variance weighted (IVW) method was employed as the primary statistical approach to assess the causal relationship between gut microbiota and sarcopenia. It is generally considered the most accurate method for estimating causality if there is no clear evidence of directional pleiotropy (*p* for MR-Egger intercept > .05). In cases where there was not enough evidence of heterogeneity among the selected instruments (*p* for MR heterogeneity > .05), a random-effects model was applied; otherwise, a fixed-effects model was assumed. Additionally, the weighted median method was performed, which can generate valid causal estimates when at least 50% of the selected instruments are valid SNPs. The MR Pleiotropy RESidual Sum and Outlier (MR-PRESSO) test assessed pleiotropy and detected outliers. Subsequently, the IVW method was repeated.

### Sensitivity Analyses

To validate our results, we performed a set of robustness checks. Cochran’s *Q* statistic was utilized to assess heterogeneity among the instrumental variables. Furthermore, to detect horizontal pleiotropy, we also employed 4 MR methods (MR-Egger, weighted median, simple mode, weighted mode) and MR-PRESSO to examine the relationship between gut microbiota, metabolites, and the risk of WP, ALM, and GS. By combining these methods, we aimed to ensure the validity and reliability of our research findings to determine the potential causal relationship between gut microbiota, metabolites, and sarcopenia. All of the above analysis was done using the TwoSampleMR package (version 0.5.6) in R (version 4.3.0).

### Ethical Approval and Consent for Participation

This study relies on publicly accessible data sets from GWAS and does not involve the use of individual-level data. Each study included in every GWAS was approved by the relevant Institutional Review Board, and prior informed consent was obtained from participants or their designated representatives (such as caregivers, legal guardians, or appointed proxies).

## Results

### Causal Estimates of Genetically Predicted Gut Microbiota on Sarcopenia

**Figure 2.**
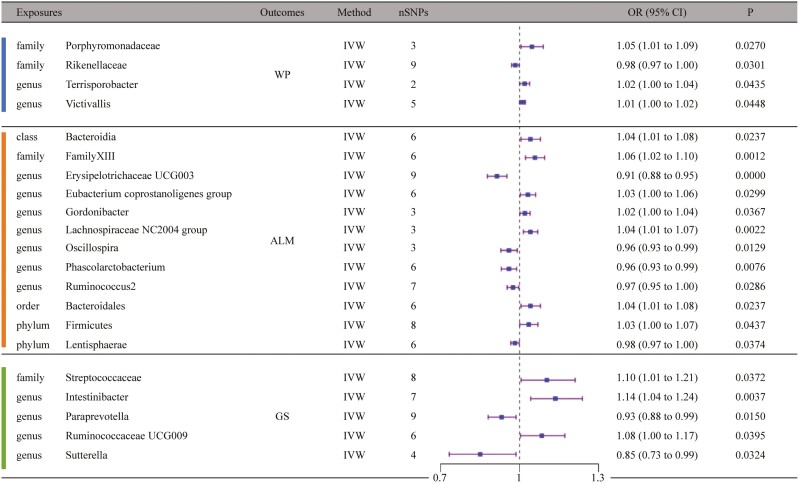
Mendelian randomization results of causal effects between gut microbiota and sarcopenia risk. ALM = appendicular lean mass; CI = confidence interval; GS = grip strength; OR = odds ratio; SNP = single-nucleotide polymorphism; WP = walking pace.

Following MR analysis, we found that family *Porphyromonadaceae* (odds ratio [OR] = 1.047, 95% confidence interval **[**CI] = 1.005–1.090, *p* = 2.70E-02, IVW), family *Rikenellaceae* (OR = 0.984, 95% CI = 0.970–0.998, *p* = 3.01E-02, IVW), genus *Terrisporobacter* (OR = 1.019, 95% CI = 1.001–1.038, *p* = 4.35E-02, IVW), and genus *Victivallis* (OR = 1.011, 95% CI = 1.000–1.022, *p* = 4.48E-02, IVW) were causally associated with WP. And for ALM, we found class *Bacteroidia* (OR = 1.042, 95% CI = 1.005–1.079, *p* = 2.37E-02, IVW), family *FamilyXIII* (OR = 1.058, 95% CI = 1.023–1.095, *p* = 1.16E-03, IVW), genus *Erysipelotrichaceae UCG003* (OR = 0.914, 95% CI = 0.878–0.952, *p* = 1.71E-05, IVW), genus *Eubacterium coprostanoligenes group* (OR = 1.032, 95% CI = 1.003–1.061, *p* = 2.99E-02, IVW), genus *Gordonibacter* (OR = 1.020, 95% CI = 1.001–1.040, *p* = 3.67E-02, IVW), genus *LachnospiraceaeNC2004group* (OR = 1.042, 95% CI = 1.015–1.069, *p* = 2.22E-03, IVW), genus *Oscillospira* (OR = 0.960, 95% CI = 0.929–0.991, *p* = 1.29E-02, IVW), genus *Phascolarctobacterium* (OR = 0.960, 95% CI = 0.931–0.989, *p* = 7.59E-03, IVW), genus *Ruminococcus2* (OR = 0.975, 95% CI = 0.953–0.997, *p* = 2.86E-02, IVW), order *Bacteroidales* (OR = 1.042, 95% CI = 1.005–1.079, *p* = 2.37E-02, IVW), phylum *Firmicutes* (OR = 1.035, 95% CI = 1.001–1.070, *p* = 4.37E-02, IVW), and phylum *Lentisphaerae* (OR = 0.983, 95% CI = 0.967–0.999, *p* = 3.74E-02, IVW) were causally associated with it. Furthermore, we found that family *Streptococcaceae* (OR = 1.104, 95% CI = 1.006–1.211, *p* = 3.72E-02, IVW), genus *Intestinibacter* (OR = 1.136, 95% CI = 1.042–1.239, *p* = 3.67E-03, IVW), genus *Paraprevotella* (OR = 0.932, 95% CI = 0.881–0.986, *p* = 1.50E-02, IVW), genus *Ruminococcaceae UCG009* (OR = 1.085, 95% CI = 1.004–1.172, *p* = 3.95E-02, IVW), genus *Sutterella* (OR = 0.850, 95% CI = 0.733–0.986, *p* = 3.24E-02, IVW) were causally associated with GS. In the sensitivity analysis, at least 1 method showed that family *FamilyXIII*, genus *Erysipelotrichaceae UCG003*, genus *LachnospiraceaeNC2004group*, genus *Oscillospira*, genus *Ruminococcus2*, phylum *Lentisphaerae*, genus *Intestinibacter*, and genus *Sutterella* had the same direction as IVW, and the *p* value was statistically significant (*p* < .05; Figure 2; Supplementary Table 4).

### Causal Estimates of Genetically Predicted Gut Microbiota-Derived Metabolites on Sarcopenia

**Figure 3.**
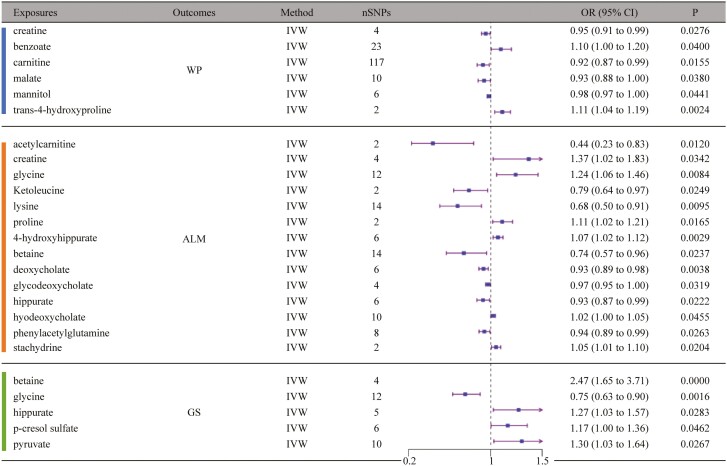
Mendelian randomization results of causal effects between gut microbiota-derived metabolites and sarcopenia risk. ALM = appendicular lean mass; CI = confidence interval; GS = grip strength; OR = odds ratio; SNP = single-nucleotide polymorphism; WP = walking pace.

Following MR analysis, we found that creatine (OR = 0.952, 95% CI = 0.911–0.995, *p* = 2.76E-02, IVW), benzoate (OR = 1.098, 95% CI = 1.004–1.201, *p* = 4.00E-02, IVW), carnitine (OR = 0.924, 95% CI = 0.867–0.985, *p* = 1.55E-02, IVW), malate (OR = 0.935, 95% CI = 0.877–0.996, *p* = 3.80E-02, IVW), mannitol (OR = 0.982, 95% CI = 0.965–1.000, *p* = 4.41E-02, IVW), and trans-4-hydroxyproline (OR = 1.113, 95% CI = 1.039–1.192, *p* = 2.39E-03, IVW) were causally associated with WP. And for ALM, we found that acetylcarnitine (OR = 0.437, 95% CI = 0.229–0.834, *p* = 1.20E-02, IVW), creatine (OR = 1.369, 95% CI = 1.024–1.832, *p* = 3.42E-02, IVW), glycine (OR = 1.243, 95% CI = 1.057–1.460, *p* = 8.35E-03, IVW), ketoleucine (OR = 0.788, 95% CI = 0.640–0.970, *p* = 2.49E-02, IVW), lysine (OR = 0.677, 95% CI = 0.504–0.909, *p* = 9.54E-03, IVW), proline (OR = 1.112, 95% CI = 1.020–1.213, *p* = 1.65E-02, IVW), 4-hydroxyhippurate (OR = 1.070, 95% CI = 1.023–1.119, *p* = 2.95E-03, IVW), betaine (OR = 0.739, 95% CI = 0.568–0.960, *p* = 2.37E-02, IVW), deoxycholate (OR = 0.930, 95% CI = 0.885–0.977, *p* = 3.75E-03, IVW), glycodeoxycholate (OR = 0.974, 95% CI = 0.951–0.998, *p* = 3.19E-02, IVW), hippurate (OR = 0.925, 95% CI = 0.866–0.989, *p* = 2.22E-02, IVW), hyodeoxycholate (OR = 1.023, 95% CI = 1.000–1.046, *p* = 4.55E-02, IVW), phenylacetylglutamine (OR = 0.938, 95% CI = 0.887–0.992, *p* = 2.63E-02, IVW), and stachydrine (OR = 1.051, 95% CI = 1.008–1.097, *p* = 2.04E-02, IVW) were causally associated with it. Furthermore, we found that betaine (OR = 2.473, 95% CI = 1.648–3.711, *p* = 1.23E-05, IVW), glycine (OR = 0.752, 95% CI = 0.630–0.897, *p* = 1.57E-03, IVW), hippurate (OR = 1.271, 95% CI = 1.026–1.575, *p* = 2.83E-02, IVW), p-cresol sulfate (OR = 1.166, 95% CI = 1.003–1.356, *p* = 4.62E-02, IVW), and pyruvate (OR = 1.302, 95% CI = 1.031–1.645, *p* = 2.67E-02, IVW) were causally associated with GS. In the sensitivity analysis, at least 1 method showed that creatine and benzoate for WP, glycine, lysine, betaine, deoxycholate, and glycodeoxycholate for ALM, as well as betaine and glycine for GS, had the same direction as IVW, and the *p* value was statistically significant (*p* < .05; Figure 3; Supplementary Table 5).

## Discussion

MR could be designed using existing large-scale GWAS data, overcoming the limitations of confounding factors that may bias traditional observational studies (22). Compared to case–control and cohort studies, MR provides a higher level of evidence, reflecting the lifelong impact of risk factors (23). The strengths of this study include utilizing the largest and most recent summary data from GWAS on gut microbiota and sarcopenia to investigate the unclear relationship of the gut–muscle axis. The HMDB, which is the largest and most comprehensive database of human metabolomics, was used to select metabolites derived from gut microbiota (15). The substantial sample size ensures the statistical power of the study results. Conducting high-quality MR analysis hinges on rigorous quality control of GWAS data. We employed LDSC as a method for data quality control, which helps distinguish inflation of statistical quantities in GWAS summary data (20).

Using MR analysis, we obtained a significant number of gene-level predicted associations between gut microbiota abundance and the phenotype of sarcopenia, further reinforcing our previous findings (24). GS and WP can serve as metrics for assessing muscle function. We have identified a positive causal relationship between *Porphyromonadaceae*, *Terrisporobacter*, *Victivallis*, *Streptococcaceae*, *Intestinibacter*, and *Ruminococcaceae UCG009* and impaired muscle function. Meanwhile, *Rikenellaceae*, *Paraprevotella*, and *Sutterella* may act as protective factors for muscle function. Based on existing research, we have observed that while some of these microbes are not commonly mentioned in the context of sarcopenia, the majority of them are linked to fat. An observational study conducted in an elderly population in Italy showed a significant association between a higher proportion of individuals with *Porphyromonadaceae* and *Rikenellaceae* and lower visceral fat (25). Similarly, an animal experiment indicated a negative correlation between *Victivallis* and obesity and hepatic steatosis (26). A meta-analysis reveals that in obese populations, the abundance of *Streptococcaceae*, *Paraprevotella*, *Sutterella* is higher compared to nonobese individuals. The increase in visceral fat and fat infiltration in skeletal muscles caused by obesity may be important pathogenic mechanisms for sarcopenia (27). ALM can serve as metrics for assessing muscle mass. We have identified that *Bacteroidia*, *FamilyXIII*, *Eubacterium coprostanoligenes group*, *Gordonibacter*, *LachnospiraceaeNC2004group*, *Bacteroidales*, and *Firmicutes* are risk factors for muscle mass loss, while there is a positive causal relationship between *Erysipelotrichaceae UCG003*, *Oscillospira*, *Phascolarctobacterium*, *Ruminococcus2*, *Lentisphaerae*, and muscle mass. Metabolic dysfunction is considered the primary mechanism through which microbes are believed to be associated with muscle mass loss. Compared to other species, *Firmicutes* and *Bacteroidales* are closely associated with the production of short-chain fatty acids, and *Eubacterium coprostanoligenes group* is closely linked to cholesterol metabolism (28). These findings appear to further support previous research on the relationship between gut microbiota and sarcopenia. Individuals with frailty and cancer cachexia show a significant reduction in the relative abundance of *Paraprevotella* and increased *Intestinibacter* and *Ruminococcaceae* (24,29,30). An observational study found a positive correlation between the relative abundance of *Bacteroides* and the severity of sarcopenia (31). Furthermore, we have observed the microbiota that do not align with the findings of other studies. A prospective cohort research has indicated a correlation between sarcopenia and an increase in the abundance of *Sutterella* (32). Animal experiments have also suggested a close association between *Sutterella* and the aging and inflammatory phenotypes in sarcopenia (33). The role of *Sutterella* in the occurrence and progression of sarcopenia, whether as a risk factor or a compensatory factor, still requires further investigation. And we observed that, although *Ruminococcus2* and *Ruminococcaceae UCG009* both belong to the Ruminococcaceae family, they have opposite directions of risk for the occurrence of sarcopenia. This may suggest that more detailed species-level classification may be necessary for future research on microbial functions.

In fecal microbiota transplantation in clinical studies, a meta-analysis has summarized the therapeutic effects of 85 different diseases. Most of these studies have focused on gastrointestinal conditions, with limited research directly related to muscle health (34). The relationship between metabolites derived from gut microbiota and sarcopenia suggests that targeting these metabolites could be a more effective intervention strategy. We found a positive causal relationship between benzoate, trans-4-hydroxyproline, betaine, hippurate, p-cresol sulfate, and pyruvate and impaired muscle function while carnitine, creatine, glycine, malate, and mannitol are protective factors for healthy muscle function. Furthermore, we also found a positive causal relationship between 4-hydroxyhippurate, creatine, glycine, hyodeoxycholate, proline, and stachydrine and muscle mass loss, while acetylcarnitine, betaine, deoxycholate, glycodeoxycholate, hippurate, ketoleucine, lysine, and phenylacetylglutamine had negative correlations. Yang et al. employed statistical methods to identify 45 significantly different metabolites in cancer cachexia patients (35). Among them, the metabolite that overlaps with our results are 4-hydroxyproline, carnitine, glycine, malate, creatine, glycocholate, lysine, pyruvate, and phenylacetylglutamine. The recent focus of research lies in understanding how the gut microbiota regulates muscle physiology. Among these, short-chain fatty acids have been extensively studied, and other bacterial metabolites include bile acids, amino acid derivatives, and polyphenol-derived metabolites. Some studies have indicated that in cachexia patients, the reduced metabolism of most amino acids by the gut microbiota leads to amino acid loss, particularly in *Lachnospiraceae* and *Ruminococcaceae*. In turn, it has additional effects on the body, including the impact of aromatic amino acid derivatives like p-cresol and phenylacetate and branched-chain amino acids that maintain normal muscle metabolism (36,37). One of the functions of gut microbiota metabolism is to convert primary bile acids into secondary bile acids. Bile acid derivatives activate the bile acid receptor TGR5 to varying degrees, leading to skeletal muscle protein degradation, muscle mass loss, and mitochondrial dysfunction in skeletal muscles. It is glycodeoxycholate that is strongly linked to the reduction in skeletal muscle volume in nonalcoholic fatty liver disease patients (32,38). It is interesting that a study has proposed the related metabolic indicators of benzoate and hippurate as biomarkers for frailty and elderly syndrome (39). While hippurate typically increases with age, it decreases in frail individuals. Whether it has a more specific connection with muscles is still a subject for further exploration. One point that must be emphasized is that betaine, creatine, glycine, and hippurate seem to have opposing effects on muscle mass and function. This may be related to dose–response, and we need to consider pharmacology and toxicology. While they may promote an increase in muscle mass, it is essential to investigate whether they also have adverse effects on muscle function. This necessitates a careful exploration of dose parameters.

The relationship between the gut microbiome and its associated metabolome remains to be fully elucidated. Visconti et al. investigated the impact of gut microbiota on host systemic metabolism, revealing that 46% of circulating metabolites are associated with microbial taxa and their metabolic pathways. Many circulating metabolites exhibit significant heritability (40). Dodd et al. demonstrated that manipulating *C. sporogenes* at a genetic level significantly altered indolepropionic acid concentrations in mice by enhancing their ability to metabolize aromatic amino acids (41). This highlights the substantial potential of gut microbiota to modulate physiological states by regulating their derived metabolites. However, the network of relationships between gut microbiota and their associated metabolites is undoubtedly complex. The influence of gut microbiota on metabolites may extend beyond metabolic activities, affecting the transfer and absorption of metabolites by modulating intestinal barrier integrity rather than bioavailability. The high redundancy of metabolic pathways among different microbial species leads to an average sharing of 82% of microbial metabolic pathways between randomly paired unrelated individuals, with an average of 10 metabolic pathways associated with each circulating metabolite (40). The intricate cross-network relationships pose challenges in establishing causal relationships between specific microbial groups and particular metabolites. Liu et al. conducted an MR study on gut microbiota and their associated metabolites, identifying and replicating only 13 causal relationships between 12 microbes and 8 metabolic traits (42). This suggests that MR may not be the optimal method for screening causal relationships between gut microbiota and metabolites.

Alterations in the abundance of specific bacterial species in the gut of sarcopenia patients may influence clinical indicators associated with muscle mass and physical function through the combined effects of multiple metabolites, resulting in variations in various clinical outcomes (43). Existing research indicates that gut microbiota primarily regulates amino acids, short-chain fatty acids, bile acid derivatives, and anti-inflammatory metabolites, all of which affect muscle mass and function (32). Differential analysis among sarcopenia patients has revealed that key metabolic-related microbes including *Lachnospiraceae*, *Firmicutes*, *Subdolicapsulum*, *Faecalibacter*, *Eubacteria coprostanoligenes group*, *Erysipelotrichaceae UCG003*, and *Ruminococcaceae* are associated with purine metabolism, arginine and proline metabolism, alanine and glutamate metabolism, butyrate metabolism, and histidine metabolism (44). Similarly, our findings identify metabolically active microbes such as *Erysipelotrichaceae UCG003*, *Eubacterium coprostanoligenes group*, *Firmicutes*, *Lachnospiraceae*, *Ruminococcaceae*, and *Ruminococcus*, which are linked to proline, glycine, glutamate, and alanine metabolism. The *Firmicutes* and *Bacteroidetes* are considered indicators of microbial dysbiosis in assessing sarcopenia and other diseases, as they regulate short-chain fatty acids, particularly butyrate, and glucose metabolism, while also exerting anti-inflammatory effects to improve muscle mass and function (45,46). Furthermore, our results suggest a potential causal relationship between phenolic compounds and uremic toxins (4-hydroxyhippurate, acetylcarnitine, hippurate, p-cresol sulfate, and phenylacetylglutamine) with sarcopenia. This presents another promising area for investigation. According to existing research, certain microbes and metabolites identified in our study exhibit strong correlations. *Streptococcus* is a prominent producer of mannitol, while *Bacteroides* is a key bacteria involved in the production of p-cresol sulfate (47,48). And phenylacetylglutamine is predominantly derived from *Lachnospiraceae* and *Ruminococcaceae* (49). Moreover, *Sutterella* has been demonstrated to produce pro-inflammatory factors like lipopolysaccharides and play a role in age-related sarcopenia (32).

We observed that the causal association directions between certain exposure traits and different phenotypes of sarcopenia, such as betaine and glycine, may vary. Other research studies on sarcopenia have also demonstrated varied effects of exposures due to the diversity of outcome data (50). This discrepancy might be linked to the definition of sarcopenia, as we chose populations displaying anomalies in WP, ALM, and GS as representatives of the condition. Sarcopenia’s definition has undergone multiple revisions in the past decade, and as of now, there exists no universally accepted diagnostic method for sarcopenia. The challenges in diagnosis present obstacles to the collection and statistical analysis of patient information (51). Individuals with diminished GS and reduced WP may not necessarily be afflicted with sarcopenia. Furthermore, those affected by the condition may manifest varying symptoms. Exploring whether there are significant differences in the composition of gut microbiota and their metabolites among populations with different symptoms is also a direction worth investigating. While patients may experience decreased muscle strength and mass, there could be notable differences in phenotypes such as GS and WP. Overall, there indeed exists a pronounced directional association between exposure and these distinct phenotypes. Further research using more advanced designs and methods to comprehensively explore the effects of these exposures is warranted.

### Limitations

It is essential to acknowledge that this study comes with certain limitations. Firstly, the MiBioGen database includes populations from various genera, and the relationship between genes and the abundance of gut microbiota may have resulted in average outcomes by eliminating some population-specific effects. However, at least 72% of individuals in the gut microbiota GWAS cohorts are of European ancestry, and the statistical data for gut microbiota-derived metabolites and outcomes (WP, ALM, and GS) predominantly originate from individuals of European descent. Genetic variation is unevenly distributed across different ethnic or racial groups, which may restrict the generalizability of the study findings to specific ethnic populations (52). Therefore, further investigation is necessary to ascertain the applicability of our study results to specific ethnic populations. Additionally, the restricted number of SNPs employed in specific gut microbiota and metabolite MR analyses could lead to causal findings that lack robust support. It is essential to underscore that, akin to other MR studies, our research relies exclusively on genomic predictions to assess the linear causal impact of exposures on outcomes, reflecting the average effect of exposures throughout the lifespan. However, these associations may be more intricate, displaying nonlinear relationships or susceptibility to environmental influences. Future directions could involve integrating genomic predictions with clinical studies or employing more sophisticated methodologies to investigate the specific roles of the microbiota and metabolites in the pathogenesis of sarcopenia. Furthermore, validating their potential as clinical biomarkers and therapeutic targets will require rigorous testing through randomized controlled trials.

## Conclusion

The MR analysis provides compelling evidence that establishes a plausible causal nexus between gut microbiota, metabolites and susceptibility to sarcopenia. Strategically altering the abundance of gut microbiota and the concentrations of their correlated metabolites stands as a feasible approach in ameliorating the risk of sarcopenia. Overall, monitoring and modulating the gut microbiota is a preemptive strategy against the onset of sarcopenia, particularly in individuals characterized by perturbed gut microbiota and metabolite profiles.

## Supplementary Material

glae173_suppl_Supplementary_Materials

## Data Availability

The data utilized in this study were sourced from publicly available summary-level data sets. Data on sarcopenia were obtained at GWAS Catalog (https://www.ebi.ac.uk/gwas/). The summary data derived from GWAS on gut microbiota (Kurilshikov et al.) can be accessed via https://www.mibiogen.org/. The metabolomic GWAS summary statistics (Shin et al.) were downloaded from the metabolomics GWAS server (https://metabolomips.org/gwas/index.php?task=download).

## References

[1] Cruz-Jentoft AJ , Sayer AA. Sarcopenia. Lancet. 2019;393:2636–2646. 10.1016/S0140-6736(19)31138-9 31171417

[2] Yuan S , Larsson SC. Epidemiology of sarcopenia: prevalence, risk factors, and consequences. Metabolism 2023;144:155533. 10.1016/j.metabol.2023.155533 36907247

[3] Tsekoura M , Kastrinis A, Katsoulaki M, Billis E, Gliatis J. Sarcopenia and its impact on quality of life. Adv Exp Med Biol. 2017;987:213–218. 10.1007/978-3-319-57379-3_19 28971460

[4] Zhang X , Huang P, Dou Q, et al. Falls among older adults with sarcopenia dwelling in nursing home or community: a meta-analysis. Clin Nutr. 2020;39:33–39. 10.1016/j.clnu.2019.01.002 30665817

[5] Feng L , Gao Q, Hu K, et al. Prevalence and risk factors of sarcopenia in patients with diabetes: a meta-analysis. J Clin Endocrinol Metab. 2022;107:1470–1483. 10.1210/clinem/dgab884 34904651

[6] Giron M , Thomas M, Dardevet D, Chassard C, Savary-Auzeloux I. Gut microbes and muscle function: can probiotics make our muscles stronger? J Cachexia Sarcopenia Muscle. 2022;13:1460–1476. 10.1002/jcsm.12964 35278043 PMC9178375

[7] Chen LH , Chang S-S, Chang H-Y, et al. Probiotic supplementation attenuates age-related sarcopenia via the gut–muscle axis in SAMP8 mice. J Cachexia Sarcopenia Muscle. 2022;13:515–531. 10.1002/jcsm.12849 34766473 PMC8818665

[8] Fielding RA , Reeves AR, Jasuja R, Liu C, Barrett BB, Lustgarten MS. Muscle strength is increased in mice that are colonized with microbiota from high-functioning older adults. Exp Gerontol. 2019;127:110722. 10.1016/j.exger.2019.110722 31493521 PMC6823114

[9] Kang L , Li P, Wang D, Wang T, Hao D, Qu X. Alterations in intestinal microbiota diversity, composition, and function in patients with sarcopenia. Sci Rep. 2021;11:4628. 10.1038/s41598-021-84031-0 33633246 PMC7907362

[10] Hou K , Wu Z-X, Chen X-Y, et al. Microbiota in health and diseases. Signal Transduct Target Ther. 2022;7:135. 10.1038/s41392-022-00974-4 35461318 PMC9034083

[11] Zhang T , Cheng JK, Hu YM. Gut microbiota as a promising therapeutic target for age-related sarcopenia. Ageing Res Rev. 2022;81:101739. 10.1016/j.arr.2022.101739 36182084

[12] Cruz-Jentoft AJ , Bahat G, Bauer J, et al.; Writing Group for the European Working Group on Sarcopenia in Older People 2 (EWGSOP2), and the Extended Group for EWGSOP2. Sarcopenia: revised European consensus on definition and diagnosis. Age Ageing. 2019;48:16–31. 10.1093/ageing/afy169 30312372 PMC6322506

[13] Emdin CA , Khera AV, Kathiresan S. Mendelian randomization. JAMA. 2017;318:1925–1926. 10.1001/jama.2017.17219 29164242

[14] Kurilshikov A , Medina-Gomez C, Bacigalupe R, et al. Large-scale association analyses identify host factors influencing human gut microbiome composition. Nat Genet. 2021;53:156–165. 10.1038/s41588-020-00763-1 33462485 PMC8515199

[15] Wishart DS , Feunang YD, Marcu A, et al. HMDB 4.0: the human metabolome database for 2018. Nucleic Acids Res. 2018;46:D608–D617. 10.1093/nar/gkx1089 29140435 PMC5753273

[16] Shin SY , Fauman EB, Petersen A-K, et al.; Multiple Tissue Human Expression Resource (MuTHER) Consortium. An atlas of genetic influences on human blood metabolites. Nat Genet. 2014;46:543–550. 10.1038/ng.2982 24816252 PMC4064254

[17] Timmins IR , Zaccardi F, Nelson CP, Franks PW, Yates T, Dudbridge F. Genome-wide association study of self-reported walking pace suggests beneficial effects of brisk walking on health and survival. Commun Biol. 2020;3:634. 10.1038/s42003-020-01357-7 33128006 PMC7599247

[18] Pei YF , Liu Y-Z, Yang X-L, et al. The genetic architecture of appendicular lean mass characterized by association analysis in the UK Biobank study. Commun Biol. 2020;3:608. 10.1038/s42003-020-01334-0 33097823 PMC7585446

[19] Jones G , Trajanoska K, Santanasto AJ, et al. Genome-wide meta-analysis of muscle weakness identifies 15 susceptibility loci in older men and women. Nat Commun. 2021;12:654. 10.1038/s41467-021-20918-w 33510174 PMC7844411

[20] Bulik-Sullivan BK , Loh P-R, Finucane HK, et al.; Schizophrenia Working Group of the Psychiatric Genomics Consortium. LD score regression distinguishes confounding from polygenicity in genome-wide association studies. Nat Genet. 2015;47:291–295. 10.1038/ng.3211 25642630 PMC4495769

[21] Burgess S , Thompson SG. Mendelian Randomization: Methods for Using Genetic Variants in Causal Estimation. CRC Press; 2015. 10.1111/rssa.12343

[22] Sanderson E , Glymour MM, Holmes MV, et al. Mendelian randomization. Nat Rev Methods Primers. 2022;2:6. 10.1038/s43586-021-00092-5 37325194 PMC7614635

[23] Arsenault BJ. From the garden to the clinic: how Mendelian randomization is shaping up atherosclerotic cardiovascular disease prevention strategies. Eur Heart J. 2022;43:4447–4449. 10.1093/eurheartj/ehac394 35869924

[24] Wang Y , Li Y, Bo L, et al. Progress of linking gut microbiota and musculoskeletal health: casualty, mechanisms, and translational values. Gut Microbes. 2023;15:2263207. 10.1080/19490976.2023.2263207 37800576 PMC10561578

[25] Tavella T , Rampelli S, Guidarelli G, et al. Elevated gut microbiome abundance of Christensenellaceae, Porphyromonadaceae and Rikenellaceae is associated with reduced visceral adipose tissue and healthier metabolic profile in Italian elderly. Gut Microbes. 2021;13:1–19. 10.1080/19490976.2021.1880221 PMC7889099 33557667

[26] Rodriguez J , Hiel S, Neyrinck AM, et al. Discovery of the gut microbial signature driving the efficacy of prebiotic intervention in obese patients. Gut. 2020;69:1975–1987. 10.1136/gutjnl-2019-319726 32041744 PMC7569399

[27] Li CW , Yu K, Shyh-Chang N, et al. Pathogenesis of sarcopenia and the relationship with fat mass: descriptive review. J Cachexia Sarcopenia Muscle. 2022;13:781–794. 10.1002/jcsm.12901 35106971 PMC8977978

[28] de Vos WM , Tilg H, Van Hul M, Cani PD. Gut microbiome and health: mechanistic insights. Gut. 2022;71:1020–1032. 10.1136/gutjnl-2021-326789 35105664 PMC8995832

[29] Jackson MA , Jeffery IB, Beaumont M, et al. Signatures of early frailty in the gut microbiota. Genome Med. 2016;8:8. 10.1186/s13073-016-0262-7 26822992 PMC4731918

[30] Zhang L , Liao J, Chen Q, et al. Characterization of the gut microbiota in frail elderly patients. Aging Clin Exp Res. 2020;32:2001–2011. 10.1007/s40520-019-01385-2 31656031

[31] Wang Y , Zhang Y, Lane NE, et al. Population-based metagenomics analysis reveals altered gut microbiome in sarcopenia: data from the Xiangya Sarcopenia Study. J Cachexia Sarcopenia Muscle. 2022;13:2340–2351. 10.1002/jcsm.13037 35851765 PMC9530518

[32] Aliwa B , Horvath A, Traub J, et al. Altered gut microbiome, bile acid composition and metabolome in sarcopenia in liver cirrhosis. J Cachexia Sarcopenia Muscle. 2023;14:2676–2691. 10.1002/jcsm.13342 37767786 PMC10751428

[33] Siddharth J , Chakrabarti A, Pannérec A, et al. Aging and sarcopenia associate with specific interactions between gut microbes, serum biomarkers and host physiology in rats. Aging (Albany NY) 2017;9:1698–1720, 10.18632/aging.101262 28783713 PMC5559170

[34] Wang Y , Zhang S, Borody TJ, Zhang F. Encyclopedia of fecal microbiota transplantation: a review of effectiveness in the treatment of 85 diseases. Chin Med J (Engl). 2022;135:1927–1939. 10.1097/CM9.0000000000002339 36103991 PMC9746749

[35] Yang QJ , Zhao J-R, Hao J, et al. Serum and urine metabolomics study reveals a distinct diagnostic model for cancer cachexia. J Cachexia Sarcopenia Muscle. 2018;9:71–85. 10.1002/jcsm.12246 29152916 PMC5803608

[36] Le Couteur DG , Solon-Biet SM, Cogger VC, et al. Branched chain amino acids, aging and age-related health. Ageing Res Rev. 2020;64:101198. 10.1016/j.arr.2020.101198 33132154

[37] Pötgens SA , Thibaut MM, Joudiou N, et al. Multi-compartment metabolomics and metagenomics reveal major hepatic and intestinal disturbances in cancer cachectic mice. J Cachexia Sarcopenia Muscle. 2021;12:456–475. 10.1002/jcsm.12684 33599103 PMC8061360

[38] Mancin L , Wu GD, Paoli A. Gut microbiota–bile acid–skeletal muscle axis. Trends Microbiol. 2023;31:254–269. 10.1016/j.tim.2022.10.003 36319506

[39] De Simone G , Balducci C, Forloni G, Pastorelli R, Brunelli L. Hippuric acid: could became a barometer for frailty and geriatric syndromes? Ageing Res Rev. 2021;72:101466. 10.1016/j.arr.2021.101466 34560280

[40] Visconti A , Le Roy CI, Rosa F, et al. Interplay between the human gut microbiome and host metabolism. Nat Commun. 2019;10:4505. 10.1038/s41467-019-12476-z 31582752 PMC6776654

[41] Dodd D , Spitzer MH, Van Treuren W, et al. A gut bacterial pathway metabolizes aromatic amino acids into nine circulating metabolites. Nature. 2017;551:648–652. 10.1038/nature24661 29168502 PMC5850949

[42] Liu X , Tong X, Zou Y, et al. Mendelian randomization analyses support causal relationships between blood metabolites and the gut microbiome. Nat Genet. 2022;54:52–61. 10.1038/s41588-021-00968-y 34980918

[43] He Y , Cui W, Fang T, Zhang Z, Zeng M. Metabolites of the gut microbiota may serve as precise diagnostic markers for sarcopenia in the elderly. Front Microbiol. 2023;14:1301805. 10.3389/fmicb.2023.1301805 38188577 PMC10768011

[44] Zhou J , Liu J, Lin Q, et al. Characteristics of the gut microbiome and metabolic profile in elderly patients with sarcopenia. Front Pharmacol. 2023;14:1279448. 10.3389/fphar.2023.1279448 38026977 PMC10654747

[45] Stojanov S , Berlec A, Štrukelj B. The influence of probiotics on the *Firmicutes/Bacteroidetes* ratio in the treatment of obesity and inflammatory bowel disease. Microorganisms 2020;8:1715. 10.3390/microorganisms8111715 33139627 PMC7692443

[46] Han DS , Wu W-K, Liu P-Y, et al. Differences in the gut microbiome and reduced fecal butyrate in elders with low skeletal muscle mass. Clin Nutr. 2022;41:1491–1500. 10.1016/j.clnu.2022.05.008 35667265

[47] Zhang M , Gu L, Cheng C, et al. Recent advances in microbial production of mannitol: utilization of low-cost substrates, strain development and regulation strategies. World J Microbiol Biotechnol. 2018;34:41. 10.1007/s11274-018-2425-8 29480337

[48] Sivsammye G , Sims HV. Presumptive identification of *Clostridium difficile* by detection of p-cresol in prepared peptone yeast glucose broth supplemented with p-hydroxyphenylacetic acid. J Clin Microbiol. 1990;28:1851–1853. 10.1128/jcm.28.8.1851-1853.1990 2394806 PMC268058

[49] Barrios C , Beaumont M, Pallister T, et al. Gut–microbiota–metabolite axis in early renal function decline. PLoS One. 2015;10:e0134311. 10.1371/journal.pone.0134311 26241311 PMC4524635

[50] Hetherington-Rauth M , Johnson E, Migliavacca E, et al. Nutrient metabolites associated with low D3Cr muscle mass, strength, and physical performance in older men. J Gerontol A Biol Sci Med Sci. 2024;79:glae131. 10.1093/gerona/glae131 37694554 PMC10809040

[51] Sayer AA , Cruz-Jentoft A. Sarcopenia definition, diagnosis and treatment: consensus is growing. Age Ageing. 2022;51:afac220. 10.1093/ageing/afac220 36273495 PMC9588427

[52] Davey Smith G , Hemani G. Mendelian randomization: genetic anchors for causal inference in epidemiological studies. Hum Mol Genet. 2014;23:R89–R98. 10.1093/hmg/ddu328 25064373 PMC4170722

